# A commentary on: “A 12-year population-based study of freezing of gait in Parkinson's disease”

**DOI:** 10.3389/fnagi.2015.00106

**Published:** 2015-06-02

**Authors:** Jacob J. Crouse, Ahmed A. Moustafa

**Affiliations:** ^1^School of Social Sciences and Psychology, University of Western SydneySydney, NSW, Australia; ^2^Marcs Institute for Brain and Behaviour, University of Western SydneySydney, NSW, Australia

**Keywords:** Parkinson's disease, freezing of gait (FOG), dopamine

A recent study by Forsaa et al. (2015) details the prospective assessments of a population-based Parkinson's disease (PD) cohort, investigating the long-term progression and associated risk factors of freezing of gait (FOG). Building on previous work examining the progression of psychosis in PD (Forsaa et al., 2010), the authors followed a cohort of 232 PD patients, assessing both motor and non-motor disease features including FOG, severity of parkinsonism, motor complications, psychotic symptoms, and cognitive impairment at 4 and 8 years, and annually thereafter.

In this study, Forsaa et al. (2015) utilized a prospective longitudinal research design, allowing for investigating and predicting symptom progression. This predictive capability is of importance to intervention and therapy, as FOG is associated with reduced quality of life (Moore et al., 2007; Walton et al., 2015), increased risk of falls and subsequent medical complications (Bloem et al., 2004), caregiver-burden (Schrag et al., 2006), and self-initiated social isolation (Bloem et al., 2004).

Forsaa et al. (2015) suggest that many PD patients may develop FOG during their disease course. This finding aids in remediating a discrepancy in the “freezing” literature, in relation to the highly variable prevalence rates of FOG in PD patients, with past research reporting variable rates between 31 and 87% (Hely et al., 2005; Macht et al., 2007; López et al., 2010; Auyeung et al., 2012; Perez-Lloret et al., 2014). Forsaa et al. (2015) report a FOG point prevalence rate of 25% at the commencement of the study, 75% at the 8-year follow-up, and 63% at the end of the study. This variability in the literature may result from previous overreliance on cross-sectional research, as well as dropout rates stemming from non-PD related comorbidities and increased mortality in PD patients displaying FOG.

As shown by Forsaa et al's (2015) long-term prospective follow-up design, repeated assessments of disease features and the use of robust statistical analyses, including logistic regression models and generalized estimating equations, the risk factors and concomitant features of FOG were explored in isolation from one another. The analyses revealed a significant relationship between motor fluctuations and FOG, as non-“freezers” presenting with motor fluctuations at the commencement of the study had more than three times the likelihood of developing FOG during the 12-year follow-up period, as compared with patients without motor fluctuations at baseline. Interestingly, dyskinesias were associated with a statistically non-significant (but still greater than 60%) reduced risk of incidence of FOG during follow-up, a finding that needs further confirmation, as suggested by Forsaa et al. (2015).

Currently, complete understanding of the pathophysiology of dyskinesias and motor fluctuations remains elusive. However, both appear to be associated with the degree of dopamine depletion and the use of levodopa (L-Dopa) (Linazasoro, 2007; Calabresi et al., 2008; Antonini et al., 2010), with approximately 50–80% of PD patients who receive L-Dopa for more than 5–10 years developing such complications (Marsden and Parkes, 1977; Rajput et al., 2002). There is mounting evidence that such motor complications might be somewhat related to the short-half life of the drug, as well as its potential to induce pulsatile stimulation of dopamine receptors. Further, treatment strategies utilizing continuous stimulation of dopamine receptors often result in reduced motor complications (Olanow et al., 2004; Antonini et al., 2010). While more research needs to be conducted, Forsaa et al's (2015) results of differential risk for FOG between dyskinesias and motor fluctuation suggest that the two may affect the basal ganglia and other brain areas differently.

The pathophysiology of FOG has been suggested to involve both dopaminergic and nondopaminergic networks in cortical, subcortical and brainstem regions (Shine et al., 2011), as evidenced by experimental and neuroimaging studies investigating the triggering of freezing episodes by visual, cognitive, and attentional tasks, as well as other environmental cues (Yogev-Seligmann et al., 2008; Almeida and Lebold, 2010; Cowie et al., 2010, 2012; Snijders et al., 2010; Matar et al., 2014; Plotnik et al., 2014). In support of the above, Forsaa et al. (2015) found that the severity of postural instability and gait disturbances (PIGD), as well as psychotic symptoms, each of which are thought to relate to extrastriatal pathology (Williams-Gray et al., 2006; Grimbergen et al., 2009), were independently associated with the occurrence of FOG during the 12-year study period.

Forsaa et al. identified several limitations to their research. First, the lack of neuropsychological data collected on subjects might have allowed for attentional dysfunctions and deficits in executive function to slip through unrecognized. Also, long intervals between follow-ups during the first eight years were identified as another limitation, one that was however remedied from the 8-year mark onward. During the initial 8 years, 136 subjects died, in which important data may have been missed. Another limitation is the authors' method of FOG assessment, which entailed utilizing a single item from the Unified Parkinson's Disease Rating Scale (UPDRS). This is limiting in that it is a fairly crude measure of “freezing,” which may not allow for identifying of subtler aspects of gait. However, until recently, more expansive questionnaires detailing in-depth descriptions of FOG were unavailable (Nieuwboer et al., 2009), but the utility and sensitivity of the New Freezing of Gait Questionnaire is still an issue of contention (Shine et al., 2012).

Given the high frequency of FOG in the PD population and its inherent negative consequences, more research into the pathophysiology, treatment and prevention of this symptom is necessary. Forsaa et al. (2015) demonstrate the utility of a population-based, longitudinal design in understanding a progressive disease like PD, offering new insights into potential predictive risk factors for FOG, and adds to the evidence-based conceptualization of PD not as the classical “disease of dopamine,” but as a complex, multi-network, multi-neurotransmitter system disease.

## Conflict of interest statement

The authors declare that the research was conducted in the absence of any commercial or financial relationships that could be construed as a potential conflict of interest.
